# Pre-Operative Physical Function Predicts Post-Operative Skeletal Muscle Gene Expression in Old Mice

**DOI:** 10.1093/geroni/igab046.3024

**Published:** 2021-12-17

**Authors:** Samantha Asche-Godin, Lauren Harlow, Zachary Graham, Weihua Huang, Charles Mobbs, Christopher Cardozo, Fred Ko

**Affiliations:** 1 Icahn School of Medicine at Mount Sinai/James J. Peters VA Medical Center, Bronx, New York, United States; 2 James J. Peters VA Medical Center, Bronx, New York, United States; 3 Birmingham VA Medical Center, Birmingham, Alabama, United States; 4 New York Medical College, Valhalla, New York, United States; 5 Icahn School of Medicine at Mount Sinai, new york, New York, United States

## Abstract

In older adults, pre-operative physical function predicts post-operative outcomes. The biological mechanisms underlying vulnerability to physical decline remain poorly understood. Using a mouse model of laparotomy, we sought to identify biological correlates of post-operative function. 24-month-old male C57BL/6N mice were categorized as high functioning (HF) or low functioning (LF) based on pre-operative performance on the accelerating rotarod. On post-operative days (POD) 2 and 4, LF mice had lower rotarod latency to fall times than HF mice did. Forelimb grip strength was reduced after laparotomy in both HF and LF groups on POD 1 and 3 and did not differ significantly between these groups. Whole transcriptome sequencing analysis (RNAseq) of soleus muscles collected on POD 5 showed 224 and 228 differentially expressed genes (DEGs) for HF and LF, respectively, compared to their respective controls. Only 21 DEGs were observed in both groups, including Pparα, Fst and Pla2g15. Such changes may be hallmarks of the post-surgical response in aging. Pathway analysis of DEGs using Ingenuity Pathway Analysis software (Qiagen) revealed one pathway common to HF and LF (osteoarthritis) whereas activation of GP6 signaling and apoptosis signaling was observed in HF and inhibition of PPARα/RXR activation and PPARα signaling was noted in LF. We conclude that pre-operative performance on the accelerating rotarod correlates with differences in skeletal muscle gene expression, which may contribute to the differences in functional outcomes post-operatively in HF and LF mice. Further studies are needed to delineate the roles of these signaling pathways in physical resilience to surgery.

